# Can a Good Theory Be Built Using Bad Ingredients?

**DOI:** 10.1007/s42113-024-00220-w

**Published:** 2024-09-13

**Authors:** Sarahanne M. Field, Leonhard Volz, Artem Kaznatcheev, Noah van Dongen

**Affiliations:** 1 University of Groningen, Groningen, Netherlands; 2 University of Amsterdam, Amsterdam, Netherlands; 3 Utrecht University, Utrecht, Netherlands

**Keywords:** Metascience, Theory, Replication, Reproducibility

## Abstract

The replication crisis threatens to seriously impact theory development in the cognitive, behavioral, and social sciences. We canvas three desiderata of scientific theories (explanation, prediction, and unification) and argue that the extent to which failures of replication prove problematic depends on the primary purpose of a theory. If the aim is to explain how nature works, then accuracy—and thus replicability—of the findings on which the theory is built is essential. If the aim is to predict outcomes, then replicability of findings from which the predictive model or theory is built is only important as far as it affects the reliability and accuracy of the predictions. If the aim is to unify and organize disparate findings, then the replicability of findings plays a non-essential role. The result is that a multifaceted and nuanced perspective is required to assess the value of replicability and the need for replication studies. Specifying a theory’s purpose and background commitments should clarify the debate on replication and contribute to better theory development in the cognitive, behavioral, and social sciences.

## Introduction

The past decade has brought with it increasing doubt over the credibility of many empirical findings that are seminal to many disciplines within the social sciences. Those findings, once thought to be robust, failed to materialize in replication studies conducted under rigorous conditions (e.g., Open Science Collaboration, 2015). These concerns about reproducibility have led to renewed attention to replication, igniting debate about the value and purpose of replication studies, especially in the current methodological and epistemological milieu of the social sciences.^1^

One question that remains open, to the best of our knowledge, concerns how low reproducibility might impact scientific theorizing. Indeed, as van Rooij and Baggio (2021) suppose, “psychological scientists may believe in the need to build large collections of robust, replicable, uncontested effects before even thinking about starting to build theories.”^2^ While there is variation in the “recipes” by which scientific endeavors proceed, the core ingredients used tend to be hypothesis formation, the development of expected consequences, and tests of those expectations against observations (Potochnik, 2019). Additionally, and more recently popularized, preregistration and the registered report format are ingredients that might be used to help boost reproducibility.^3^ These ingredients would be less useful, however, without some way of systematizing them, and so scientific theories are needed to play their familiar roles in the advancement of scientific knowledge across disciplines and the development of our understanding of the world around us. In cases where these ingredients “go bad,” though, can scientists still generate good theories? We contend that the answer depends, at least in part, on the scientists’ aims and philosophical orientation. We take theories to be large-scale systems of ideas about natural phenomena, which are more general and more elaborate units of knowledge than individual hypotheses, and with much more evidence to support them (see Guest and Martin 2021 for a useful graph and discussion on how elements like theory, hypotheses and data relate to one another), and we take them to have at least three main desiderata: Scientific theories afford predictions of future events, provide explanations of natural phenomena, and unify diverse content. Scientists judge particular theories as “good” or “bad” based on how well they (1) explain the “why” and “how” of natural phenomena, (2) predict future events and responses to interventions, or (3) unify diverse and seemingly disconnected observations into coherent frameworks.

### Cooking up Scientific Theories

Intuitively, producing a high-quality theory *should* require good inputs. For the sake of literary flair, consider a loosely applied culinary analogy: not just anything can be thrown together to get a good flambéed bombe Alaska or Swiss meringue. Certain ingredients and technical skills, as well as patience, are needed. Constructing good theories would also seem to require that the elements and parts have certain qualities. This analogy raises the question of whether mounting numbers of failed replications indicate that the pantries of many scientists are ill-stocked.

Specifically, the three theoretical desiderata (explanation, prediction, unification) might express three aspects of science (basic science, applied science, scientific culture) and can be somewhat connected to three philosophies of science (realism, instrumentalism, constructivism).^4^ The degree to which irreplicable findings are problematic depends on the scientists’ goals and philosophy of science, and thus the function of scientific theories that they find most important. And, because scientists are seldom monomaniacal about the function of theories, there is likely to be at least some value in theories based on bad ingredients.

In the next section, we begin by characterizing what we mean by “bad ingredients” and provide a short overview of the replication crisis. The subsequent three sections are each dedicated to one of the functions that scientific theories can fulfill: prediction, explanation, and unification. In each section, the function will be addressed in terms of scientific practice, philosophy of science, and to what extent bad ingredients can be a problem.

### The Replication Crisis: A Pantry of Potentially Spoiled Ingredients

As with other scientific fields, behavioral science has experienced periods of sanguinity; the past decade, however, has been characterized by a period of doubt, known, among other names, as the reproducibility crisis, or crisis of confidence in psychology. Skepticism is rife, and many researchers doubt the trustworthiness of many of the foundational effects of their fields (Baker, 2016). Ioannidis’s 2005 article sparked the disquiet, in which he claimed that most published findings are false. His claims were explosive at the time of publication, and while his argument was only theoretical (and has been the subject of refutation itself; see Goodman & Greenland (2007)), the concerns it reflected seem largely to have been borne out by later empirical research.

In 2015, *Science* published a landmark attempt by 270 researchers to estimate the replicability of 100 articles from three high-impact psychology journals. Reportedly, only a third of these were successfully replicated, a finding that can be interpreted as indicating serious structural flaws in the psychology literature. Doubts about the integrity of individual researchers also abounded following reports of fraud and misconduct (see, e.g., Verfaellie and McGwin, 2011; John et al. 2012) or researchers’ refusal to share their study data (Vanpaemel et al., 2015).

Although discussions about the potential value of replication studies have appeared in the literature for decades (see, e.g., Rosenthal, 1990), interest in and support of replication has soared in connection with concerns about the trustworthiness of research in the social sciences. The Dutch Research Council (NWO) alone pledged 3 million euros to replication studies; in the US, the Laura & John Arnold Foundation put more than 15 million dollars into encouraging and supporting replication in the scientific community. Journals such as the *Journal of Trial and Error* and the *Journal in Support of the Null Hypothesis* have been established to actively encourage reporting mistakes, flaws, and null findings. The research literature has become peppered with replication studies (individual as well as wide-scale) and other metaresearch studies that deal with the topic of replication (e.g., selecting which studies are most in need of replication: Field et al., 2019; Pittelkow et al., 2021, 2023).

With the increasing interest in replications comes indications of which phenomena are reproducible and which are not.^5^ Such reproducibility might give us a sense of what ingredients might be good to use, and which we might want to consider leaving out.

### The Value of Replication in Science

With this renewed interest in replication, there is debate as to its value to science. How much information (and of what kind) do replications provide? Certainly, the key objective of conducting replications is to improve or verify scientific knowledge about a given phenomenon. Another function of replication is to “allay doubts about specific results or about the proper execution of a previous study” (pg. 27 of Royal Netherlands Academy of Arts and Sciences, 2018). Clemens (2017) presents a framework for explaining a replication in terms of its specific information value: replications that are methodologically similar to the original can provide information about unwitting errors, type-I error, or potential QRP. Those that vary the methodology, on the other hand, can provide information about the generalisability of the original finding, and the various conditions under which it can be successfully replicated. While one could argue that this framework is somewhat outdated by now, as well as being too simplistic or naive, it gives a sense of how one can (and how researchers have and do) use replication studies for different purposes.

More recently, Derksen and Morawski (2022) reject the dichotomous conceptualization of replication types, arguing: “that opposition of direct and conceptual replication, and the way they are commonly associated with emphases on permanence and variability, respectively, is unhelpful.” They develop an alternative perspective to the discussion of different replication types that they dub the “enactment perspective,” in which different replication methods can potentially produce a variety of scientific “realities.”

### Spoiled Ingredients: The Value of Phenomena that Cannot be Reproduced

How problematic is it that some findings cannot be replicated? Perspectives of scholars recorded in the scientific literature run the gamut, from framing replication as being critical—a cornerstone of the scientific method (Simons, 2014)—to being critical about the value of replication (de Rijcke & Penders, 2018; Leonelli, 2018). Both perspectives may have merit for understanding good theory, albeit within a certain context and philosophy of science.

Scientific theories aim to explain phenomena, predict future events and patterns, and unify or structure our knowledge. These purposes can be approximately linked to aspects of science: basic science, applied science, and the socio-cultural nature of science, respectively. These purposes can also be viewed from some positions in the philosophy of science: realism, instrumentalism, and constructivism, respectively. For each purpose, the value of replicability is different.

In the next three sections, we outline how the value of results that do not replicate can depend on the purpose of science that one finds most important, the aspect of science one values, and the philosophy of science that one holds. Before we go further, however, please note the following important caveats and assumptions regarding philosophies and purposes, as they underlie our arguments in what follows.

1. These philosophies of science are in practice quite broad and come in many varieties. They do not map one-to-one on the three purposes, and we do not wish to claim otherwise. For instance, van Fraassen’s anti-realism concerns itself with explanation in terms of empirical adequacy (Van Fraassen, 1980), and constructivist can care about prediction (e.g., Hennig, 2023). Instead, we assert that there is a general match between each purpose and philosophy.
2. These purposes by themselves are not what makes theories scientific. For instance, other types of ideas and non-scientific theories could, at least in principle, also offer consistent and accurate predictions. This does not detract from the purpose of prediction that one might have.
3. The purposes as we delineate them are epistemic in nature. They do not also constitute a moral virtue and can even be at odds with them. For instance, the theory of eugenics attempted to unify elements from, among others, psychology, biology, and anthropology, though it is universally condemned for its unethical implications and the crimes committed in its name (e.g., Guest, 2024).
4. We acknowledge that prediction, explanation, and unification also have their roles in causal reasoning. It is not our intention to limit our perspective to causal inference only, and we refrain from engaging with this literature for several reasons. First, not all (causal) explanations allow for meaningful predictions (e.g., earthquakes). Some explanations are, for instance, (partially) constitutive or mathematical in nature. Second, not all explanations are causal. Third, (causal) explanations can unify, but do not *have* to do so (e.g., smoking causing lung cancer).

## Three Purposes of Scientific Theory

### Purpose 1: Explanation

Our first purpose of scientific theories is *explanation*; in particular, we refer to the explanations of regularities, structure, phenomena, and events in nature, societies, or within and between people. Explanations do not occur in a vacuum. From a theoretical background (e.g., assertions of patterns, properties, and processes of nature), a description is given of why or how the phenomenon in question has or will come about. Scientific theories play a critical role in our society and technological development because they offer precise explanations to enable understanding of the world around us (e.g., the theory of general relativity underpins GPS satellites).

In its most rudimentary form, an explanation is *an accounting of something by something else*. Philosophical perspectives on (scientific) explanation (e.g., Hempel and Oppenheim, 1948; Hempel, 1965; Salmon, 1984; Woodward, 2003; Schupbach and Sprenger, 2011) offer rules and evaluations about how or why a phenomenon or event (i.e, the *explanandum*) follow from other events, phenomena, first principles, mathematical regularities, statistical relations, causal structures, etc. (i.e., the *explanans*). An explanation boils down to accepting the explanans as given and demonstrating the process of the explanandum systematically resulting from these explanans. A conceptual distinction between basic science and applied science can be useful when talking about scientific practice. The pursuit of explanation is closely intertwined with the domain of basic science, which aims to uncover the fundamental laws and principles that govern the natural world, irrespective of its practical applications.

The aim of explaining how nature works typically assumes that there is something to explain and thus that there is an objective reality whose mechanisms we aim to uncover (related to scientific realism; Chakravartty, 2017). Scientific theories, according to this view, aim to accurately describe the nature of the world as it truly exists—explanations for why things are the way they are—by mirroring its mechanisms in some abstracted and/or idealized form. This endeavor of uncovering and understanding the underlying structure of the world ties the purpose of explanation to a generally realist view on the structures of theories.

From this realist perspective, scientific theories must accurately describe the world as it exists to be of explanatory value. Their consequences must be testable and replicable through observation and experimentation. Replicability of research results, either used for developing or testing the theory, is thus critical when it comes to the purpose of explaining phenomena. Replications ensure that scientific findings are not random occurrences or anomalies but provide a sturdy and reliable foundation of evidence on which an explanatory theory can be built.^6^ Replicability also allows other researchers to confirm or refute scientific claims and provides a means of testing the validity of scientific theories. In short, without replicability, scientific findings would lack the necessary level of rigor and stability to be deemed accurate (i.e., corresponding to the underlying structure), thereby undermining the explanations provided by the theory.

### Purpose 2: Prediction

Ideally, as van Rooij and Baggio (2021) stress, “we do not construct theories just to explain effects” (p. 682). There are other purposes to be spoken of. For instance, another purpose of scientific theories is to precisely and consistently predict novel events. Theories posit a model from which predictions can be derived and tested against empirical results. The most famous example is the theory of quantum electrodynamics that predicts the magnetic moment of the electron to an error margin of roughly ten parts in a billion ($10^{-8}$). For many scientists, predictions are the bread and butter of their work: We formulate hypotheses, test these against data, and reject or retain our theory based on the agreement between the data we predicted and the data we got.

Typically, a prediction is a conjecture of something that is yet to happen (i.e., *novel events*) or a retrodiction of a past event that is not part of the theory (i.e., *use-novel events*). Consistent, accurate, and precise predictions safeguard against merely accommodating what has been observed (e.g., Bacon, 1878[1620]; Popper, 1963; Zahar, 1973; Maher, 1988). A theory being able to predict means that novel or use-novel events conjectured by the theory are observed within an acceptable margin of error.

Thus, it is not necessary to know why or how the predicted result came about as long as predictions pan out with an acceptable degree of consistency and accuracy. This is a perspective similar to the philosophical view of *instrumentalism*. Introduced by Duhem (2016), instrumentalism contends that scientific theories are just tools for predicting observations. As we can never know nature directly, it might be vastly different than what our sensory experiences and measurement instruments tell us and the best we can hope for is that our theories connect to some regularities of nature that allows us to make accurate and precise predictions for the time being.

Predictions have clear practical utility. They inform us about the expected outcome of an action/intervention or what might happen if we do not intervene. In this sense, the focus on prediction can be approximately tied to applied science. Explanation and understanding play only a secondary role in as far as they contribute to reaching the intended goals. For instance, it can be of the utmost importance to be able to predict how a patient will respond to a particular treatment (e.g., cancer treatment), which clearly outweighs the need to understand the mechanisms behind the response (e.g., the development of cancer and physiology of the tumors) if this cannot contribute to the predictive accuracy.

Having predictability as the primary concern has consequences for the need for replicable research findings. We assume that theories are constructed in part from or informed by previous findings. It is likely that the less a theory is built from replicable findings, the less likely it becomes that it gives accurate and precise predictions. However, this does not imply that all results that inform the theory need to be replicable. As long as the predictive theory functions accordingly—and prediction is what we care about—then its history or foundation should not matter. For instance, Eye Movement Desensitisation and Reprocessing (EMDR) is a therapeutic intervention for the treatment of post-traumatic stress disorder (PTSD), which predictably produces moderately successful results (e.g., McLay et al. 2016). However, Thyer et al. (2015) contest that “EMDR proponents have come up with ad hoc hypotheses to explain away unfavorable results that do not support its theory” (p. 221), which hurts the explanatory power of EMDR, though leaves the predictive efficacy unaffected. In short, predictive theories are well served by foundation with high replicability, though universal replicability is not necessary,^7^

### Purpose 3: Unification

A third purpose of scientific theories is to unify seemingly unrelated observations and phenomena. By providing a framework for understanding how different phenomena are related, scientific theories help create a unified understanding of the natural world and facilitate knowledge transfer between fields (Herfeld & Lisciandra, 2019; Kaznatcheev & Lin, 2022; Cheng et al., 2023). When different areas and phenomena within a single field (e.g., electricity and magnetism) or different fields of science (e.g., genetics and ecology) are unified under a single theory, it becomes easier for their respective researchers to collaborate and share insights. Through unification, seemingly disparate observations and phenomena can be connected and understood within a common framework. This allows for the creation of a systematic, condensed, and organized body of knowledge that can be used to inform further research.^8^ For example, the unifying principle of evolution helps organize knowledge about the diversity of life on Earth. It connects, at least, biology, genetics, and ecology through the recognition that all living organisms share a common ancestry and have evolved over time through selection pressures from their environment.

Unification highlights the social character of science and a *constructivist* philosophy of science. As seemingly unrelated phenomena are integrated under a single theoretical framework, it is important to keep in mind that this process is a socio-cultural activity that is influenced by the values, beliefs, and norms of the society in which it is practiced. These values and beliefs shape (to some extent) the questions that are asked, the methods used to answer them, and the interpretations of the results obtained (for more, see Longino, 1990; Laslett et al. 1996; Haraway, 2016; Harding, 2023). For example, the unification of electricity and magnetism under the theory of electromagnetism was not only the result of empirical observation, but was also influenced by the cultural values and beliefs of the scientists involved (Schaffer, 2011).

This social character of science also reminds us that observer-independent reality is inaccessible. What is accessible is our personal reality (i.e., the perspective of reality created by an individual through perception and interaction) and the social reality that surrounds us (i.e., a shared perspective of reality between individuals created through communication and interaction (e.g., Gergen, 2015a, b; Hennig, 2023). Our theories are actively constructed by scientists in society, rather than passively received from the environment (for more on constructivism, see Gillett, 1998). Our scientific theories might have been inspired by nature but are not necessarily true or uniquely optimal descriptions of nature.^9^

Theory’s ability to unify—to organize knowledge and bring scientists together—does not depend on its veracity,^10^ The phlogiston theory is a prime example. The theory was widely accepted in the eighteenth century and posits that all combustible materials contain a substance called phlogiston, which is released during combustion. This theory unified a wide range of observations: rusting, burning, combustion, and the release of light and heat from the latter two processes. The theory of phlogiston rested on the existence of a substance in materials called phlogiston. One of the primary hypotheses of the theory was that burning material should decrease in weight. Phlogiston, stored in the material, is released when combusted or set to rust, thus decreasing in weight proportional to the amount of phlogiston released. Scientists first thought that they indeed observed this decrease in weight, for instance, when burning wood. However, this was not consistently observed, and controlled experiments with precise measurements consistently showed that metals gained weight when rusting. While the theory was eventually refuted, the phenomena of combustion and rusting remained unified^11^ and actually paved the way for its replacement, de Lavoisier (2019) oxygen theory.

An illustrative example from psychology is Freud’s theory of the unconscious and the ego. The theory has been discredited, and the anecdotal evidence on which Freud based his theory was not replicated under a more thorough contemporary investigation. However, it is hard to overstate its contribution to clinical, developmental, and personality psychology and psychiatry (e.g., Whitebook, 2017). When the goal is unification, results on which a theory is based do not need to be replicable for the theory to be successful.

## Conclusion

In this essay, we put the value of replication in the context of several purposes that can be served by scientific theories. We asked the question of whether good theory can be derived from research whose ingredients are of questionable quality. The answer—like much in science—is that it depends. Value requires a frame of reference, a context, or a way to contrast one thing against another, and various contexts are at play when we consider replication or reproducibility. We evaluate the impact of reproducibility (or a lack thereof) on the activity of theory development from the perspective of three aims of scientific theory—prediction, explanation, and unification—and some corresponding aspects and philosophies of science.^12^ Depending on the goal one has for a scientific theory, unreproducible findings can be fundamentally problematic for theory development, practically (ir)relevant, or of minor concern. When theories play an explanatory role, the ingredients matter—unreliable studies will necessarily lead to unreliable explanations about phenomena. When theories play a predictive role, however, the quality of the studies upon which a theory is built only matters to the extent that they impact the accuracy of predictions. When unification is the goal of a theory, ingredient quality is somewhat beside the point.^13^

We expect that disagreements about the role that replication plays in science can be resolved (in part) when participants reflect on and explicate the purpose they see for scientific theories and the role that research results play in the development of such theories. For a reasonable interpretation and evaluation of theories in the light of the implications of low reproducibility in some fields, we need scientists to be transparent about their philosophies of science.^14^ Although a reliable scientific literature is generally valuable as an ideal to strive towards, it is unlikely to be *required* in all cases along the way. There is no absolute value of replication. There is only the value relative to one’s (or the community’s) philosophy of science and the aims of the related theory.

## Data Availability

No datasets were generated or analyzed during the current study.

## Footnotes

1 See, for instance, the Special Section on Replicability in Psychological Science: A Crisis of Confidence? (2012).

2 Though we emphasize here that the arguments furthered in van Rooij and Baggio ’s article are counter to some of the views, we are engaging with in this paragraph.

3 Although we also note here that plenty of arguments exist against preregistration’s value in improving reproducibility—see McDermott’s perspective essay on the topic (Toth et al., 2021; McDermott, 2022).

4 Note that we do not intend to imply that the elements on each of these triads are strongly linked to one another. Consider these triads in terms of their illustrative value and as a way to structure the text.

5 Note that we differentiate here between reproducible (by which we mean able to be replicated) and “verified” or “true.”

6 We acknowledge that observations are theory-laden. However, singular observations and the general phenomena they evidence are typically not wedded to a single theory nor particularly wedded to the theory that explains it. For instance, the phenomenon that candles burn for a short period before they supper out in an airtight space was known before the phlogiston theory and fits both the phlogiston theory and oxygen theory.

7 Formal modeling even suggests that universal replicability is not always possible in such cases, “[b]ecause irreproducibility, when formally defined, appears to be an inherent property of the scientific process rather than a meaningful scientific objective to pursue” (p. 20; Buzbas et al. 2023).

8 In some cases, as argued by van Rooij and Baggio (2021), such candidate theories must be built to help guide us away from fixating on “the majority of the (infinitely possible) effects [that] are irrelevant” and to instead find the “many of the relevant effects (sentences) [that] may never be discovered by chance” (p. 683). At this early stage of the theoretical cycle, the quality of ingredients (basic or secondary effects to be explained) going into the theory is beside the point, since the theorist just aims to start the iterative process of formalizing a theory that meets basic theoretical plausibility constraints. Only after theories have been sufficiently developed and refined for us to “determine which effects they imply” (p. 683), do we turn to explanatory or predictive purposes and start to worry about the quality of ingredients. This stage of unification before explanation or prediction could happen quickly within a given scientist/team, or more slowly across a scientific community.

9 In some cases, our personal and social reality actively misrepresenting an inaccessible observer-independent reality can produce a (social) interface through which to interact with the world in ways that are more beneficial to us as both individuals and groups (Hoffman, 2009; Kaznatcheev et al., 2014).

10 By veracity, we are loosely referring here to the correspondence theory of truth: a statement or belief is true if it corresponds to the facts (see Glanzberg 2023).

11 We make a distinction here. On the one hand, there are phenomena that a theory, such as the phlogiston theory, tries to explain. These phenomena were typically known before the theory was proposed, replicated consistently, and enjoyed an existence (semi)independently from the theory. An example of such a phenomenon is a burning candle in an airtight space sputtering out after a period of time proportional to the volume of that space. On the other hand, we have putative claims (hypotheses) on which the theory rests, such as the existence of an invisible substance (i.e., phlogiston) that has mass and takes up space.

12 Note that we do not claim that the aims we discuss are theories’ only or most relevant aims nor that the philosophies of science we connected to these aims are the only or best suited for the job.

13 This last case of unreproducible findings being of minor concern in the case of theories that have the goal of unification can be particularly shocking. It can be especially jarring if one is after demarcations of science from pseudoscience (or other ways of knowing) via a single criterion like reproducibility. But we are not after such demarcations in this paper. In future work, it would be interesting to contrast theory in fields like history (that are not traditionally viewed as scientific) or parapsychology (that is viewed as pseudoscience) to theories aimed at unification in science.

14 In stressing the importance of philosophy of science to practicing scientists, we are in agreement with Laplane and colleagues’ (2019) conclusion that “...Modern science without philosophy will run up against a wall: the deluge of data within each field will make interpretation more and more difficult, neglect of breadth and history will further splinter and separate scientific sub disciplines, and the emphasis on methods and empirical results will drive shallower and shallower training of students” (p. 3951).
